# Social alignment matters: Following pandemic guidelines is associated with better wellbeing

**DOI:** 10.1186/s12889-022-13130-y

**Published:** 2022-05-03

**Authors:** Bahar Tunçgenç, Martha Newson, Justin Sulik, Yi Zhao, Guillaume Dezecache, Ophelia Deroy, Marwa El Zein

**Affiliations:** 1 Department of Psychology, Nottingham Trent University, London, UK; 2 Institute of Cognitive & Evolutionary Anthropology, University of Oxford, Oxford, UK; 3 School of Anthropology and Conservation, University of Kent, Kent, UK; 4 Cognition, Values and Behaviour, Ludwig Maximilian University, Munich, Germany; 5 School of Medicine, Indiana University, Bloomington, Indiana, USA; 6 LAPSCO, Université Clermont Auvergne, CNRS, Clermont-Ferrand, France; 7 Munich Center for Neuroscience, Ludwig-Maximilian University, Munich, Germany; 8 Institute of Philosophy, School of Advanced Study, University of London, London, UK; 9 Institute of Cognitive Neuroscience, University College London, London, UK; 10 Adaptive Rationality Center, Max-Planck for Human Development, Berlin, Germany

**Keywords:** Wellbeing, Mental health, Pandemic adherence, Social distancing, Social alignment, Covid-19 lockdown

## Abstract

**Background:**

In response to the Covid-19 pandemic, most countries implemented physical distancing measures. Many mental health experts warned that through increasing social isolation and anxiety, these measures could negatively affect psychosocial wellbeing. However, socially aligning with others by adhering to these measures may also be beneficial for wellbeing.

**Methods:**

We examined these two contrasting hypotheses using cross-national survey data (N = 6675) collected fortnightly from participants in 115 countries over 3 months at the beginning of the pandemic. Participants reported their wellbeing, perceptions of how vulnerable they were to Covid-19 (i.e., high risk of infection) and how much they, and others in their social circle and country, were adhering to the distancing measures.

**Results:**

Linear mixed-effects models showed that being a woman, having lower educational attainment, living alone and perceived high vulnerability to Covid-19 were risk factors for poorer wellbeing. Being young (18–25) was associated with lower wellbeing, but longitudinal analyses showed that young people’s wellbeing improved over 3 months. In contrast to widespread views that physical distancing measures negatively affect wellbeing, results showed that following the guidelines was positively associated with wellbeing even for people in high-risk groups.

**Conclusions:**

These findings provide an important counterpart to the idea that pandemic containment measures such as physical distancing negatively impacted wellbeing unequivocally. Despite the overall burden of the pandemic on psychosocial wellbeing, social alignment with others can still contribute to positive wellbeing. The pandemic has manifested our propensity to adapt to challenges, particularly highlighting how social alignment can forge resilience.

**Supplementary Information:**

The online version contains supplementary material available at 10.1186/s12889-022-13130-y.

## Background

The Covid-19 global health crisis threatens to also be a mental health and wellbeing crisis. Vulnerabilities, however, are not evenly distributed, especially with wellbeing being a complex construct. Positive wellbeing is marked by subjective feelings of happiness, life satisfaction and having a sense of purpose in connection with the social environment one lives in [1, 2]. As such, a wide range of individual, physical, social and demographic factors combine to influence wellbeing both positively and negatively  – important to bear in mind when much emphasis has been on the negative changes associated with the pandemic. Moreover, wellbeing can fluctuate over time, rendering it critical to capture longitudinal trends. In this study, we test how adherence to Covid-19 physical distancing measures is linked to wellbeing using longitudinal, global survey data collected in April-August 2020.

Studies examining wellbeing during the pandemic have primarily focused on individual and group predictors, with findings showing associations with certain demographic factors. In particular, being a woman, being a young adult, having low educational attainment and income, and having prior mental health conditions were associated with poorer wellbeing [3–6]. Meta-analytic and review studies have shown that compared to the pre-pandemic period, the public’s levels of depression and anxiety saw a small but significant increase in the first months of the pandemic, while findings remained mixed for overall wellbeing [3, 5, 6]. A closer look suggests that living alone and having less social support were associated with increased loneliness and depressive symptoms [4, 7–9], and the fear of catching the disease was associated with increased anxiety [10–12].

Early in the pandemic, The Academy of Medical Sciences reported adverse effects of the pandemic on mental health and wellbeing as the public were anxious about catching the disease and as social isolation increased because of lockdowns promoting physical distancing [13]. Experts highlighted how unnatural physical distancing measures are to human interactions and warned against potential negative effects of ‘Draconian’ lockdown measures on mental health and wellbeing [14]. In contrast to the sentiment that lockdowns and physical distancing adversely affect mental health and wellbeing, more recent research shows that more stringent pandemic measures were indeed associated with better wellbeing [15–17]. A meta-analysis covering 226,000 participants from 26 countries revealed that only public transport closures, but not stay-at-home requirements, were associated with increased anxiety [3]. These findings can be partly explained by the majority support for lockdowns given their perceived benefits for protection from the disease [18].

Here, we focus on another reason why pandemic guidelines may be associated with better wellbeing – social alignment arising from the similarity in behaviours and experiences of a group of people. Social alignment has the potential for bringing people together under a new set of behavioural norms during a period of high threat and uncertainty [5]. Sharing common experiences during challenging times (as captured in the phrase “We’re all in it together!”) is a strong catalyst for forging social alignment and cohesion [19–21], which can be rewarding [22] and lead to better wellbeing [23–26]. Can, then, pandemic guidelines boost wellbeing, in contrast to the background expectation that social isolation would have negative effects? To inform evidence-based recommendations about behavioural policies, we need to identify the demographic and pandemic-specific factors that may underlie potential unifying effects of Covid-19 guidelines on wellbeing.

As per previous studies, we predicted that certain demographic groups, i.e., young adults, women, people with lower educational attainment and people living on their own, would have poorer wellbeing (**Hypothesis 1**). Regarding pandemic-specific factors, we predicted perceived vulnerability of self and others to the disease to be associated with poorer wellbeing (**Hypothesis 2**), whereas following physical distancing guidelines to be associated with better wellbeing (**Hypothesis 3**). To probe the origins of Hypothesis 3, we further examine whether positive effects on wellbeing were due to physical protection from catching Covid-19 or due to social alignment.

## Methods

### Participants

Participants completed a longitudinal, online survey distributed through social media platforms, university and professional mailing lists, and university press releases. To reach a diverse sample, the survey was presented in 12 languages: Arabic, Bangla, German, English, Spanish, French, Hindi, Italian, Mandarin, Persian, Swedish, Turkish. First timepoint (T1) took place between 9^th^ April and 20^th^ May 2020, with the survey remaining online for five weeks in each language at T1. Subsequent timepoints (T2-T6) took place fortnightly after T1.

The number of people participating in the survey per timepoint was *n* = 6675 at T1, *n* = 2105 at T2, *n* = 1832 at T3, *n* = 1504 at T4, *n* = 1253 at T5 and *n* = 1169 at T6. Participants opting out of certain questions led to some missing data (i.e., if they had no one in their close circle: *n* = 1199 at T1; or if they did not reveal their country of residence: *n* = 41 at T1). Table 1 shows the sociodemographic characteristics of the study population.

#### Ethics statement

The study was approved by the ethics committee of the University of Nottingham School of Psychology. All participation was in line with the General Data Protection Regulation (GDPR) and the Helsinki Declaration of 1975, as revised in 2008. Participants provided written informed consent and were assigned an anonymous ID for analysis.

#### Patient and public involvement

The public were consulted, engaged and informed at all stages of the research wherever possible. Due to time constraints while setting up the survey, we could not formally involve the public in a focus group. Instead, a convenience sample of members of the public living in a diverse range of countries (i.e., Bangladesh, England, France, Germany, India, Iran, Italy, Spain, Sweden, Turkey and USA) were consulted to provide informal feedback on our survey items, namely those assessing demographics, vulnerability to Covid-19 and adherence to guidelines, to ensure the questions reflected pandemic experiences in their countries. In addition, the public were involved in the data collection process through both participating in the study and helping  to disseminating the survey to others. The results were shared with the public at multiple stages of the study through blog posts, social media activity and media interviews.

### Materials & Procedure

The T1 survey was longer than surveys administered at T2-T6, though the variables reported in this study were collected at all timepoints except for some demographic questions (i.e., age, gender, education). Full survey items can be found at: https://osf.io/kmxez/.

#### Demographics

Participants reported their age, gender (options: man, woman, non-binary, prefer not to say), highest educational attainment (options: no schooling completed, primary education, secondary education, university undergraduate degree, postgraduate degree), number of people in their household (dichotomised as solo vs cohabiting with others), and work/study status (dichotomised into active vs  inactive with work/study).

#### Vulnerability

Participants indicated how vulnerable to the Covid-19 disease they considered (a) themselves and (b) loved ones using continuous scales, where 1 = Not vulnerable at all, 50 = As vulnerable as an average person, and 100 = Extremely vulnerable.

#### Adherence

Participants rated how well they had been following the general advice of keeping distance from others as applied in their local area on a continuous scale, where 0 = I have not been following the advice at all, 50 = I have been following the advice exactly, and 100 = I have been doing more than what is advised. In addition, we asked people how others in their close social circle (i.e., people they would turn to for advice/comfort during challenging times) and people in their country had been following these guidelines. These items were adapted from pre-pandemic research examining normative and empirical expectations [27].

#### Wellbeing

Participants completed the 7-item short Warwick-Edinburgh Mental Wellbeing Scale (WEMWBS) considering their feelings in the past week [28]. WEMWBS measures wellbeing as a single-factor construct, comprising affective-emotional, cognitive-evaluative and psychological aspects. Since the Persian version of WEMWBS was not available, native speakers proficient in English translated and back-translated it for this survey. Short WEMWBS is a well-established scale with good content and construct validity, strong internal consistency (0.91), high test–retest reliability (0.83), and relatively low social desirability bias [28]. WEMWBS has been successfully adapted to many cultures and languages [29]. Additionally, we asked participants to rate how depressed, anxious, angry and lonely they had been feeling in the past week; analyses of these item are reported in Supplementary Materials (SM). Both WEMWBS and these mood items were answered on a 5-point Likert scale, where 1 = None of the time, 2 = Rarely, 3 = Some of the time, 4 = Often, 5 = All of the time.

#### Stringency

Using the Oxford Covid-19 Government Response Tracker (OxCGRT) dataset [30], we obtained a stringency metric, which was used as a co-variate in all of our analyses. OxCGRT recorded the stringency of a range of Covid-19 measures applied in over 180 countries (and states within the US) from public gathering restrictions and school/workplace closures to social distancing and mask mandates. Adding this variable was critical due to the high variability across countries and states in terms of the prevalence of Covid-19 [31] and the measures taken to curtail its spread. Using the timeseries data in OxCGRT, we obtained a stringency score per participant by calculating the rolling average of the overall stringency score in the participant’s region within the 14 days preceding the date of their survey completion.

### Statistical Analyses

Analyses were conducted using RStudio 1.3.959, packages car, nlme and tidyverse [32]. For each hypothesis, we report analyses examining T1 only and change over the 6 timepoints. For all analyses, linear mixed-effects models were conducted, with wellbeing as the outcome variable and the participants’ country of residence as a random effect to account for the fact that participants are nested within countries. For change over time analyses, we also included timepoints as a random effect, random slope and in interaction with the predictor variables of that model (intra-class correlation analyses in SM). Hypothesis 1 models had age (levels: split into categories by every 10 years), gender, and household as predictors. Hypotheses 2–3 models included age, gender, household status, education and the stringency of measures used in the participants’ country or state [30] as covariates. Including these covariates partially addresses the fact that our samples are not representative of the population structures. Tables S13-14 show descriptive statistics of all variables used in this study, and Fig S1 shows how key variables of wellbeing, perceived vulnerability to the disease and adherence to pandemic guidelines vary across countries.

For ease of visualisation and to account for non-linear effects, we converted continuous predictors into categorical variables depending on data spread. All findings were replicated with continuous variables (Tables S7-12). For Hypothesis 2, perceived self-vulnerability and loved ones’ vulnerability variables were categorised using a median split. For Hypothesis 3, self-adherence categories were created based on the 25% and 75% quantiles: low adherence (score < 49), medium adherence (scores 49–79), and high adherence (score > 79).

To examine social alignment, the stringency variable was categorised using a median split and two ‘compliance’ variables were created that indicate how similar people’s adherence behaviour was to the perceived adherence of their close circle’s (close circle compliance) and country’s (country compliance). For these compliance scores, we took the absolute difference between participants’ self-adherence and the perceived adherence of (a) their close circle, and (b) fellow citizens. We then categorised these compliance scores into high vs low (median split). For example, a person who strongly adheres to the distancing guidelines would have a high adherence score, yet may still have a low compliance score, if their close circle and/or fellow citizens were reported to have low adherence  to the guidelines.

Additionally, we ran the same models using an aggregate mood variable as the outcome variable, which comprises 4 custom-made mood items on anxiety, depression, loneliness and anger (Tables S16-21). Distinctly from the wellbeing scores reported here, these mood items aimed to capture unique aspects of mental health and were not derived from standardised scales.

## Results

### Demographic risk factors of poorer wellbeing

Examination of Hypothesis 1 at T1 revealed significant main effects of age (F[6,6513] = 61.02,* p* < 0.0001), gender (F[3,6513] = 12.99, *p* < 0.0001), education (*F*[1,6513] = 12.38, *p* = 0.0004), household (*F*[1,6513] = 16.99,* p* < 0.0001), and work/study status (F[1,6513] = 30.08, *p* < 0.0001) on wellbeing. As predicted, young people aged 16–24 reported poorer wellbeing compared to all other age groups, women compared to men, people living solo compared to those living with others, people with lower educational attainment compared to those with  higher educational attainment, and people active with work/study compared to those neither working nor studying (Table S1). Further, non-binary people had poorer wellbeing as compared to the reference category of men (*p* = 0.02).

**Fig. 1:**
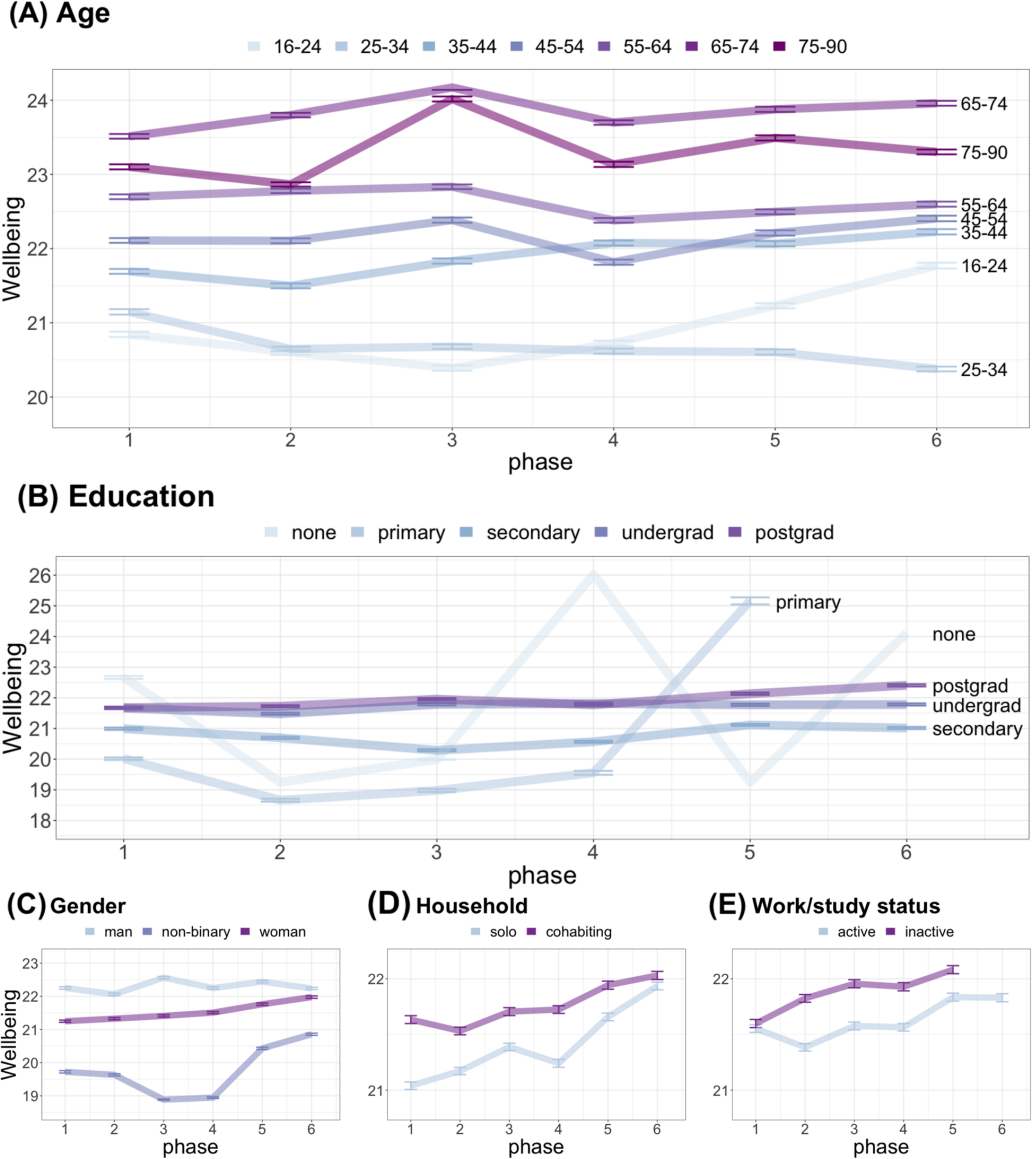
Demographic risk factor and welbeing over time. Association of wellbeing with (**A**) age, (**B**) education, (**C**) gender, (**D**) household and (**E**) work/study status across 6 timepoints. Points show the group mean and bars show the standard error of the mean

Examination of Hypothesis 1 over time (Fig. 1) revealed significant main effects of the same demographic factors (Table S2). In addition, we found that time*age interaction was significant (*F*[6,7725] = 4.46, *p* = 0.0002), but not time*gender (*F*(3,7725) = 1.30, *p* = 0.27), time*education (F(1,7725) = 0.69, *p* = 0.40), time*household (F(1,7725) = 1.56, 

*p* = 0.21) or time*work/study status (*F*(1,7725) = 0.23, *p* = 0.63). Post-hoc tests for the time*age interaction, treating time as a categorical variable to allow for non-linear changes over time, showed that wellbeing significantly improved over time only for the youngest age group of 16–24 year-olds (F(5,1242) = 2.74, *p* = 0.02; for other age groups, Table S3-4).

### Perceived disease vulnerability is linked with poorer wellbeing

**Fig. 2:**
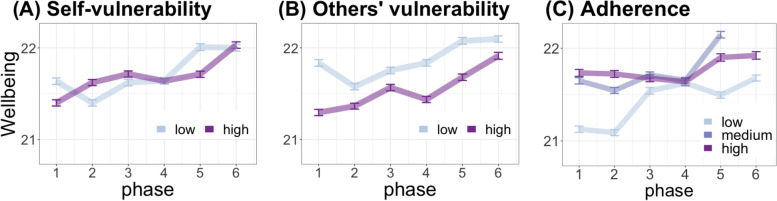
Pandemic-specific factors and welbeing over time. The associations between (**A**) self-vulnerability and wellbeing, (**B**) loved ones’ vulnerability and wellbeing, and (**C**) adherence to physical distancing guidelines and wellbeing. Dots show the group mean and bars show the standard error of the mean

Examination of Hypothesis 2 (Fig. 2) at T1 revealed significant main effects of perceived self-vulnerability (*F*(1,6516) = 23.49, β = 0.53, SE = 0.11,* p* < 0.0001) and loved ones’ vulnerability (*F*(1,6516) = 8.36, β = 0.30, SE = 0.10, *p* = 0.004), indicating that at the start of the pandemic, increased perceptions of vulnerability to the disease were associated with poorer wellbeing. Examination of Hypothesis 2 over time showed these main effects fell just above the significance threshold (self-vulnerability: *F*(1,7733) = 3.50, *p *= 0.06; loved ones’ vulnerability: *F*(1,7733) = 3.62, *p* = 0.06), and time*vulnerability interaction was not significant (self-vulnerability: *F*(1,7733) = 0.13, *p* = 0.72, loved ones’ vulnerability: *F*(1,7733) = 1.21,* p* = 0.27).

### Following pandemic distancing guidelines is linked with better wellbeing

Examination of Hypothesis 3 at T1 revealed that increased adherence to distancing guidelines was significantly associated with better wellbeing (*F*(2,6516) = 7.05, *p* = 0.0009). As compared to people displaying high adherence, those displaying low adherence had poorer wellbeing (*p* = 0.03). Importantly, adherence was positively associated with wellbeing even within demographic risk groups (Table S5). Speaking to the directionality of the found effect, a model predicting adherence (continuous values) from wellbeing (median split, categorical) was not significant at any timepoint (Table S6).

Examination of Hypothesis 3 over time revealed a similar main effect of adherence (*F*(2,7733) = 7.01, *p* = 0.0009) and a significant time*adherence interaction (*F*(2,7733) = 4.78, *p* = 0.008). Post-hoc analyses showed improved wellbeing over time for people with low (*F*(1,1490) = 4.32, β = 0.08, SE = 0.04, *p* = 0.04) and medium adherence (*F*(1,2899) = 15.09, β = 0.10, SE = 0.03, *p* = 0.0001), but not high adherence (*F*(1,1372) = 2.29, β = 0.06, SE = 0.04, *p* = 0.13).

Utilising other variables in our dataset, we probed why adherence was positively linked to wellbeing. If following the distancing guidelines were linked to better wellbeing due to increased protection from the disease, we would expect people with higher perceived vulnerability to benefit from adherence more. To test this possibility, we repeated our Hypothesis 3 model, adding adherence*vulnerability interactions. Both interaction terms were insignificant (self-vulnerability: F(2,6510) = 0.99, *p* = 0.37, loved ones’ vulnerability: *F*(2,6510) = 1.42, *p* = 0.24), indicating that adherence is positively linked to wellbeing irrespective of disease vulnerability.

**Fig. 3:**
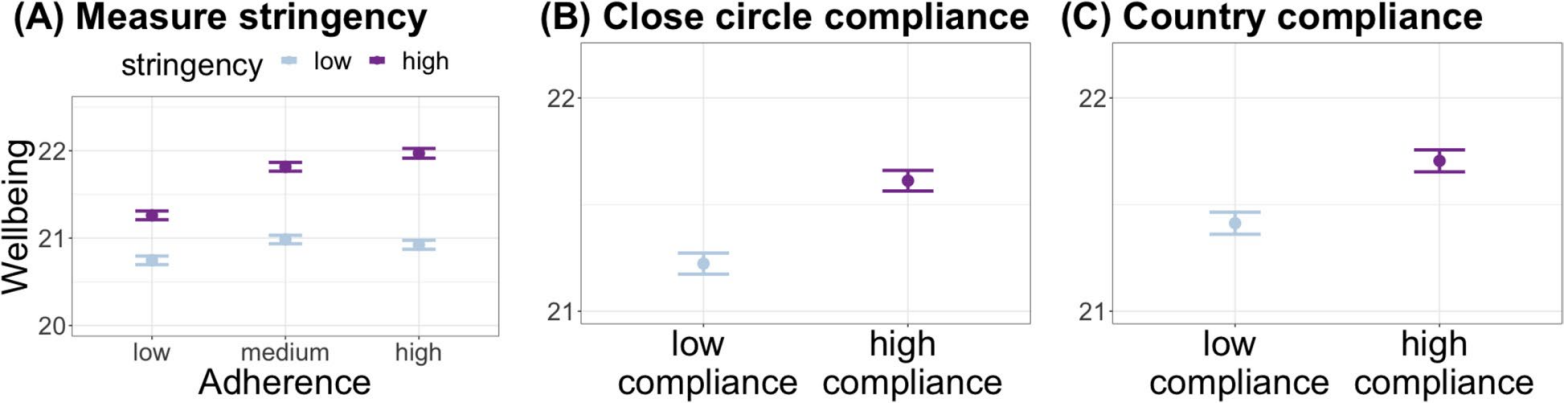
Social alignment, adherence to pandemic guidelines and welbeing. (**A**) Adherence is more strongly linked to better wellbeing when more stringent measures are implemented. Behaving more similarly (i.e., high compliance) to (**B**) one’s close circle, and (**C**) fellow citizens in one’s country are associated with better wellbeing. Dots show the group mean and bars show the standard error of the mean

Next, we conducted two analyses to assess how adherence may be positively linked to wellbeing through increased social alignment: (i) when pandemic containment measures are objectively stricter as assessed by the OxCGRT database, making people behave in more similar ways, and (ii) when participants subjectively perceive others as adhering to the guidelines more similarly to themselves. For the first analysis, we repeated the Hypothesis 3 model by adding stringency as an interaction term with self-adherence. This interaction term was significant (*F*(2,6514) = 6.48, *p *= 0.002), showing that as stringency increased, adherence was more positively associated with wellbeing (low vs medium adherence: β = 0.29, SE = 0.14,* p* = 0.04, low vs high adherence: β = 0.57, SE = 0.16,* p* = 0.0003; Fig. 3a).

For the second analysis, we re-ran the Hypothesis 3 model twice, replacing self-adherence scores with variables indicating participants’ compliance with their close circle and with people in their country. The results (Fig. 3b-c) revealed that high compliers (i.e., people who behaved more similarly to others) had better wellbeing than low compliers (close circle compliance:* F*(1,5337) = 4.67, *p* = 0.03, β = 0.007, SE = 0.003, country compliance: *F*(1,6517) = 8.03, *p* = 0.005, β = 0.006, SE = 0.002). These findings support the idea that adherence to guidelines may be linked to better wellbeing because of increased social alignment.

## Discussion

Using a cross-national sample, this study shows that key demographic factors and perceived vulnerability to the disease were risk factors for wellbeing, while social alignment attained through following pandemic guidelines was associated with better wellbeing. Our longitudinal analyses show that these effects were consistent throughout the 3-month period during which data were collected.

Beyond replicating known demographic risk factors, this study adds important extensions to our current knowledge. First, despite young adults aged 16–24 having the poorest wellbeing of all age groups, there was a significant improvement in welbeing  over time, which was specific to this age group. This shift may be due to an initial disruption to these young people’s study and work lives [33], but suggests a better adaptation to the new circumstances, and potential for resilience. Second, our sample included individuals who identified as non-binary, a group often unrepresented in psychosocial health studies [34]. We found that throughout the 3-month study period, non-binary individuals had the lowest wellbeing of all gender categories we presented, with improvements occurring over time. Future research is needed to address other demographic risk factors for which we did not collect data, such as having low household income or belonging to an ethnic minority, as well as important characteristics of participants’ history, such as pre-existing mental health conditions.

Regarding pandemic-specific factors, we found that while perceived vulnerability to the disease was associated with poorer wellbeing, higher adherence to social and physical distancing guidelines was associated with better wellbeing. A supplementary model confirmed that adherence contributed to better wellbeing, and not that people with better wellbeing simply adhered to the guidelines more (Table S6).

The reasons why adherence to pandemic guidelines could benefit wellbeing are not straightforward. Our additional analyses indicate social alignment, and not protection from the disease, is the driving force. Our data revealed that, over time, following the guidelines exactly as given – not doing more or less than required – was best for one’s wellbeing. Other pandemic research examining coordinated helping behaviour [35] and stringency of lockdown measures [3] similarly suggested that conditions leading to increased behavioural alignment among people can be positively associated with wellbeing. Replications of the positive link between social alignment and better wellbeing is an important avenue for future research in crisis contexts such as the pandemic. Using similar proxy measures of alignment as in this study, future studies could use satellite and population-level data to examine whether higher degrees of behavioural alignment in communities is linked to better mental health. In a broader context, our findings demonstrating the beneficial wellbeing effects of social alignment are in line with the social cure model, which emphasises the importance of shared social identities for promoting physical and mental health, both during [36, 37] and beyond the pandemic [38].

The mean wellbeing values obtained in this study (Table S15) were similar to those found in other UK-based pandemic studies using WEMWBS [39, 40]. This is important to note in consideration that this study did not have a representative sample, an issue partially addressed also by controlling for important risk factors for wellbeing, such as age and gender, in the analyses. A qualitative comparison shows that the wellbeing scores obtained in our sample (M = 21.6) were lower and considerably more distributed towards the lower end of the scale as compared to those reported in pre-pandemic studies. These previous community sample studies using the same measure (short WEMWBS) revealed scores ranging from 23.6 in the UK [41] to 25.4 and 26.4 in Denmark and Iceland, respectively [42]. Studies using the long version of WEMWBS, whose scores can be halved to roughly equate the short WEMWBS scores, revealed scores ranging from 46.1 to 59.9 in Australia [43], Austria [44], France [45, 46], Germany [46], Italy [47], Spain [48], the UK [28] and the USA [46]. These comparisons indicate that the 7-item short WEMWBS used in this study captured pandemic-induced decreases in welbeing, a point important to consider by public health professionals and policymakers. In addition, our study provides support for the use of short WEMWBS in future research examining rapid changes in mental health and wellbeing.

It may initially seem counterintuitive that pandemic guidelines can be positive for wellbeing beyond their disease prevention function, given that these measures also meant less face-to-face contact with loved ones [9, 49]. In our sample, the median size of people’s close social circle (n = 4) was comparable to that found in previous, pre-pandemic studies [50, 51]. Thus, it seems that following distancing guidelines did not necessarily mean social isolation, which may explain why, contrary to mental health experts’ cautioning, adherence to guidelines was not associated with poorer wellbeing. Importantly, our findings do not invalidate concerns over Covid-19 measures potentially increasing the likelihood of specific mental illnesses, such as anxiety or depressive disorders. Although mental health and wellbeing are closely related, people with a mental illness can have good wellbeing, and people without a mental illness can have poor wellbeing [52].

Overall, we highlight that following the distancing guidelines promoted better wellbeing through increased social alignment. Social alignment was marked by people behaving more similarly to others around them, sharing experiences and responsibility. Pre-pandemic literature shows that social alignment through behavioural similarity and shared experiences is associated with increased social support, cohesion and wellbeing [19, 24]. Further, people are more likely to relinquish individual responsibility about decisions in threatening and uncertain situations, which is associated with reduced stress [53]. Hence, following the guidelines may have boosted wellbeing via reducing the burden of individual responsibility during the pandemic.

## Conclusions

This study asserts that adhering to a challenging change in behavioural norms, here distancing measures, is associated with better wellbeing irrespective of people’s perceived vulnerability to the disease or other demographic risk factors for wellbeing. Thus, social alignment can be a powerful tool in high-threat and uncertain situations like the Covid-19 pandemic. We recommend that policymakers and public health officials emphasise the required actions as collective actions in local and national communities. Such an approach is key for promoting both adherence to guidelines and people’s wellbeing, which are essential for long-term coping and social cohesion.

**Table 1:** Sociodemographic characteristics of the study population at T1

		**T1**	**T2**	**T3**	**T4**	**T5**	**T6**
**Age**	**16–24 years**	1505	378	325	231	173	156
	**25–34 years**	2093	606	484	361	289	270
	**35–44 years**	1310	405	363	319	259	208
	**45–54 years**	812	303	260	229	189	192
	**55–64 years**	633	255	258	237	207	207
	**65–74 years**	264	116	107	97	108	105
	**75–90 years**	58	31	30	26	25	28
**Gender**	**man**	2204	524	454	370	314	291
	**woman**	4356	1532	1344	1107	918	859
	**non-binary**	59	26	14	13	8	7
	**not disclosed**	56	12	15	10	10	9
**Household**	**solo**	818	335	293	259	224	210
	**cohabiting**	5857	1768	1539	1245	1029	959
**Education**	**none**	13	1	2	1	1	1
	**primary**	18	3	2	2	2	1
	**secondary**	1071	286	244	176	136	142
	**undergraduate**	3096	843	719	578	472	436
	**postgrad**	2477	961	860	783	639	586
**Work/study status**	**active**	5532	1678	1456	1189	965	884
	**inactive**	1143	427	376	315	288	285
	**TOTAL**	**6675**	**2105**	**1832**	**1504**	**1253**	**1169**

## Supplementary Information

**Additional file 1.** 

## Data Availability

The dataset generated and analysed during the current study is publicly available in the Open Science Framework repository, https://osf.io/ke5yn/.
