# Supplementary material for: Social alignment matters: Following pandemic guidelines is associated with better wellbeing
Source: BMC Public Health. 2022 May 3;22:821. doi: 10.1186/s12889-022-13130-y (PMC9060841; doi:10.1186/s12889-022-13130-y)
Supplement: Supplementary file 1 — Additional file 1. [file 12889_2022_13130_MOESM1_ESM.docx]

**Supplementary Materials for**

**Following pandemic guidelines is associated with better wellbeing: Findings from a cross-national sample**

Table of Contents

[1 Detailed results about the analyses reported in the main text 2](#_Toc77851535)

[1.1 Intra-class correlation analysis 2](#_Toc77851536)

[1.2 Key variables across countries 3](#_Toc77851537)

[1.3 Full model outputs 4](#_Toc77851538)

[2 Replication of all analyses using continuous variables 13](#_Toc77851539)

[3 Aggregate mood variable 19](#_Toc77851540)

[3.1 Descriptive statistics 19](#_Toc77851541)

[3.2 Replication of all analyses using the aggregate mood variable 22](#_Toc77851542)

# Detailed results about the analyses reported in the main text

## Intra-class correlation analysis

We conducted intra-class correlation (ICC) analysis to check whether participants from the same country displayed very similar levels of wellbeing, our main outcome variable in this study. This analysis allowed us to determine whether multi-level modelling was needed, and whether the variable time (i.e., the 6 time-points in our dataset) should have a fixed or random slope.

An unconditional means model (i.e., null model) predicting wellbeing only from the intercept revealed evidence for clustering, with a log likelihood of -37879.4 and an ICC of 0.74. Next, we ran an unconditional growth model with time as a fixed slope, which had a log likelihood of -37867.31 and an ICC of 0.74. An ANOVA test comparing these two models revealed significant difference, *p* < .0001. Next, we ran a third model, this time including time as a random slope, which had a log likelihood of -37738.23 and an ICC of 0.78. An ANOVA test comparing the second and third models revealed significant difference, indicating that the third model with time as a random slope was a better fit, *p* < .0001. Given evidence of clustering in our dataset and these model comparisons, we included time as a random effect and a random slope in our longitudinal analysis models.

## Key variables across countries

| 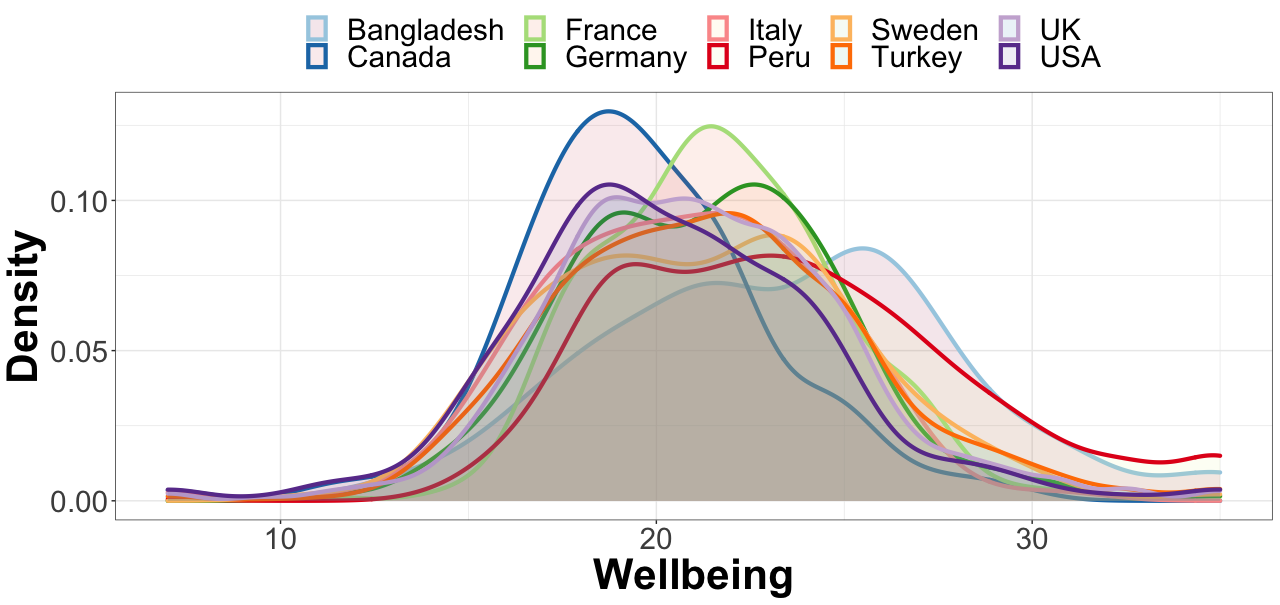 |
| --- |
| 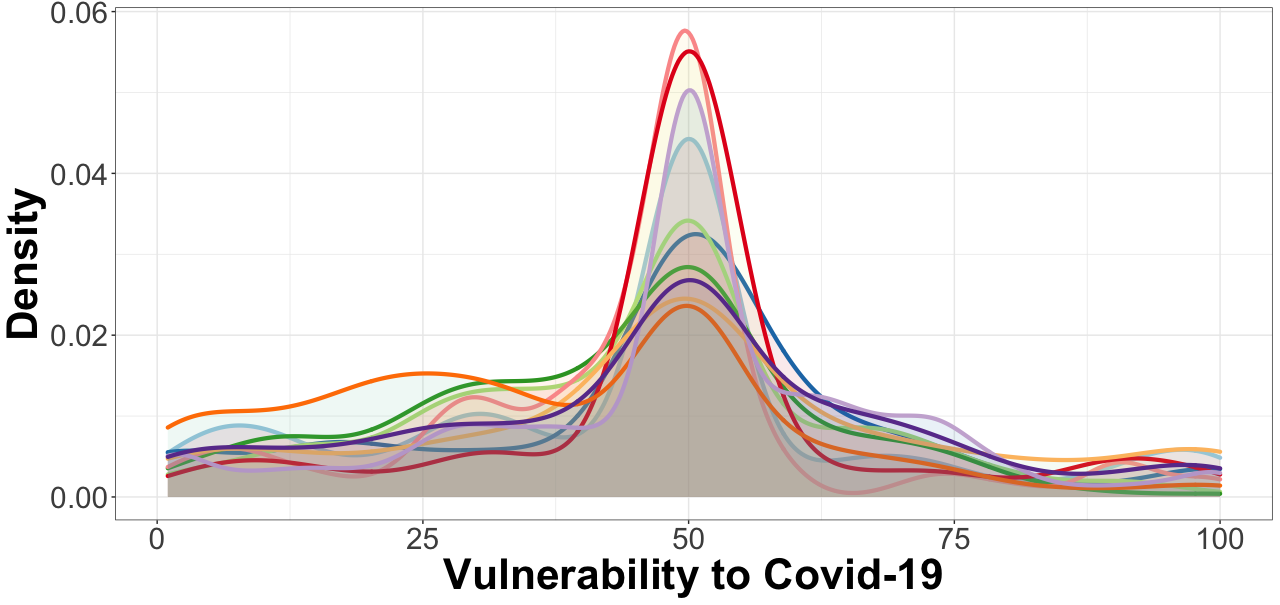 |
| 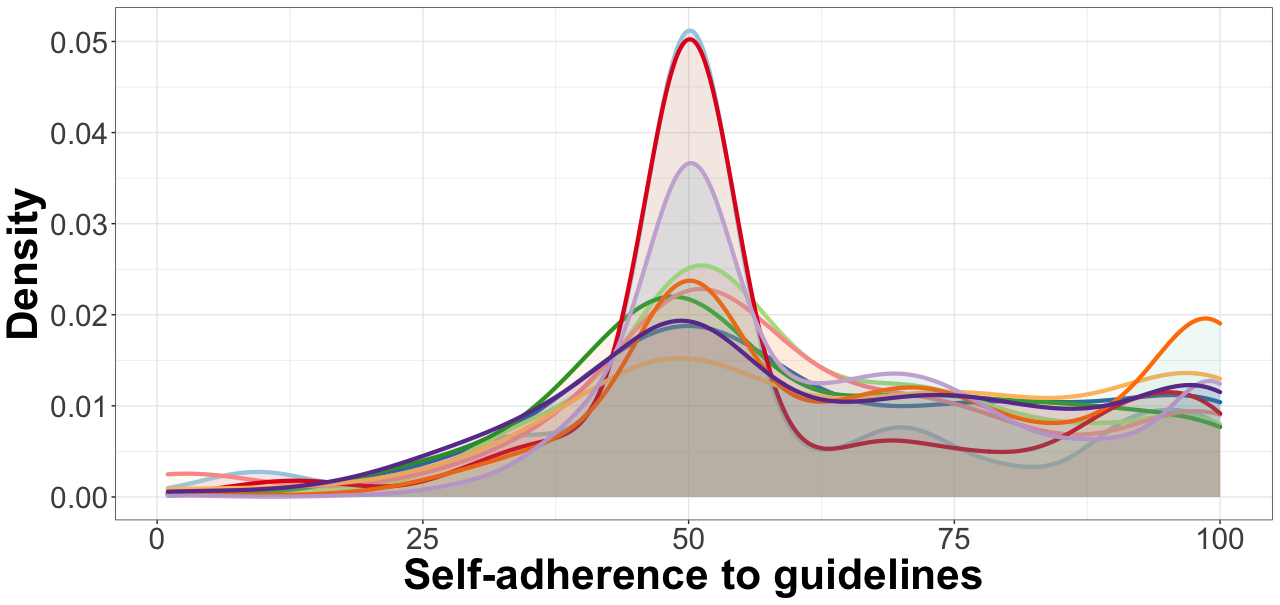 |
| **Fig. S1. Density plots showing how the countries with N > 100 sample differ in key variables** |

## Full model outputs

| *Table S1. Demographic risk factors of wellbeing at T1 (Hypothesis 1)* | | | |
| --- | --- | --- | --- |
|  | **Standardised beta** | **Std Error** | **T-test** |
| Intercept | 19.55 | .34 | *t(*6513) = 57.37, *p* < .0001 |
| Age: 16-24 vs 25-34 | 0.69 | .15 | *t(*6513) = 4.59, *p* < .0001 |
| Age: 16-24 vs 35-44 | 1.49 | .17 | *t(*6513) = 8.86, *p* < .0001 |
| Age: 16-24 vs 45-54 | 2.12 | .19 | *t(*6513) = 11.29, *p* < .0001 |
| Age: 16-24 vs 55-64 | 2.92 | .21 | *t(*6513) = 14.07, *p* < .0001 |
| Age: 16-24 vs 65-74 | 4.05 | .30 | *t(*6513) = 13.70, *p* < .0001 |
| Age: 16-24 vs 75-90 | 3.53 | .56 | *t(*6513) = 6.35, *p* < .0001 |
| Gender: men vs non-binary | -1.21 | .53 | *t(*6513) = -2.29, *p* = .02 |
| Gender: men vs not disclosed | -1.14 | .56 | *t(*6513) = -2.05, *p =* .04 |
| Gender: men vs women | -0.65 | .11 | *t(*6513) = -5.94, *p* < .0001 |
| Household: solo vs cohabiting | 0.63 | .15 | *t(*6513) = 4.12, *p* < .0001 |
| Education | 0.28 | .08 | *t(*6513) = 3.52, *p =* .0004 |
| Work/study status: active vs inactive | -0.78 | .14 | *t(*6513) = -5.48, *p* < .0001 |
| Stringency of measures | -0.001 | .13 | *t(*6513) = -0.01, *p =* .99 |

| *Table S2. Demographic risk factors of wellbeing across time-points (Hypothesis 1)* | | | | |
| --- | --- | --- | --- | --- |
|  | **Standardised beta** | **Std Error** | **T-test** | **Anova test for main effects** |
| Intercept | 19.63 | .36 | *t(*7725) = 54.14, *p* < .0001 |  |
| Age: 16-24 vs 25-34 | 0.88 | .17 | *t(*6569) = 5.32, *p* < .0001 | *Age:*  *F(6,* 6569) = 56.43, *p* < .0001 |
| Age: 16-24 vs 35-44 | 1.53 | .19 | *t(*6569) = 8.24, *p* < .0001 |  |
| Age: 16-24 vs 45-54 | 2.32 | .20 | *t(*6569) = 10.92, *p* < .0001 |  |
| Age: 16-24 vs 55-64 | 3.18 | .22 | *t(*6569) = 14.22, *p* < .0001 |  |
| Age: 16-24 vs 65-74 | 4.20 | .31 | *t(*6569) = 13.45, *p* < .0001 |  |
| Age: 16-24 vs 75-90 | 3.58 | .58 | *t(*6569) = 6.23, *p* < .0001 |  |
| Gender: men vs non-binary | -1.27 | .58 | *t(*6569) = -2.18, *p* = .03 | *Gender:*  *F(3,* 6569) = 11.39, *p* < .0001 |
| Gender: men vs not disclosed | -0.99 | .61 | *t(*6569) = -1.62, *p =* .10 |  |
| Gender: men vs women | -0.67 | .12 | *t(*6569) = -5.62, *p* < .0001 |  |
| Household: solo vs cohabiting | 0.58 | .15 | *t(*7725) = 3.86 *p =* .0001 | *F(1,* 7725) = 14.87, *p* < .0001 |
| Education | 0.25 | .09 | *t(*6569) = 2.96, *p =* .003 | *F(1,* 6569) = 8.76, *p* = .003 |
| Work/study status: active vs inactive | -0.70 | .14 | *t(*7725) = -4.97, *p* < .0001 | *F(1, 7725*) = 24.70, *p* < .0001 |
| Stringency | -0.09 | .06 | *t(*7725) = -1.53, *p =* .12 | *F(1, 7725*) = 2.36, *p* = .12 |
| Time-points | 0.08 | .10 | *t(*7725) = 0.84, *p =* .40 | *F(1, 7725*) = 0.70, *p* = .40 |
|  | **Standardised beta** | **Std Error** | **T-test** | |
| Time x Age: 25-34 | -0.21 | .06 | *t(*7725) = -3.63, *p =* .0003 | |
| Time x Age: 35-44 | -0.07 | .06 | *t(*7725) = -1.13, *p* = ..26 | |
| Time x Age: 45-54 | -0.14 | .06 | *t(*7725) = -2.31, *p =* .02 | |
| Time x Age: 55-64 | -0.26 | .06 | *t(*7725) = -4.04, *p* < .0001 | |
| Time x Age: 65-74 | -0.15 | .08 | *t(*7725) = -1.76, *p =* .08 | |
| Time x Age: 75-90 | -0.02 | .13 | *t(*7725) = -0.17, *p =* .87 | |
| Time x Gender: non-binary | 0.0004 | .19 | *t(*7725) = 0.002, *p =* .99 | |
| Time x Gender: not disclosed | -0.23 | .20 | *t(*7725) = -1.16, *p* = .24 | |
| Time x Gender: woman | -0.05 | .04 | *t(*7725) = 1.39 *p* = .16 | |
| Time x Household: cohabiting | -0.05 | .04 | *t(*7725) = -1.25, *p* = .21 | |
| Time x Education | 0.02 | .03 | *t(*7725) = 0.83, *p* = .40 | |
| Time x Work/study status: inactive | -0.02 | .04 | *t(*7725) = 0.48, *p* = .63 | |

| *Table S3. Improvement in wellbeing from one age category to the next at T1* | | | | |
| --- | --- | --- | --- | --- |
| **Age groups** | **Standardised beta** | **Std Error** | **DF** | **p value** |
| 16-24 vs 25-34 | 0.69 | .15 | 6513 | < .0001 |
| 25-34 vs 35-44 | 0.80 | .14 | 6513 | < .0001 |
| 35-44 vs 45-54 | 0.63 | .18 | 6513 | .0004 |
| 45-54 vs 55-64 | 0.81 | .21 | 6513 | .0001 |
| 55-64 vs 65-74 | 1.12 | .30 | 6513 | .0002 |
| 65-74 vs 75-90 | -0.51 | .58 | 6513 | .38 |

| *Table S4. Improvement in wellbeing across time-points (T1 to T6) within each age group* | | | | |
| --- | --- | --- | --- | --- |
|  | **Standardised beta** | **Std Error** | **T-test** | **Anova test for main effects** |
| **16-24 years old** | | | | |
| T1 vs T2 | 0.27 | .17 | *t(*1242) = 1.60, *p* = .11 | *Time-points:*  *F(5,* 1242) = 2.74, *p* = .02 |
| T1 vs T3 | 0.16 | .19 | *t(*1242) = 0.85, *p* = .40 |  |
| T1 vs T4 | 0.20 | .23 | *t(*1242) = 0.87, *p* = .38 |  |
| T1 vs T5 | 0.54 | .26 | *t(*1242) = 2.06, *p* = .04 |  |
| T1 vs T6 | 0.96 | .29 | *t(*1242) = 3.36, *p* = .001 |  |
| **25-34 years old** |  |  |  |  |
| T1 vs T2 | 0.008 | .12 | *t(*1967) = 0.07, *p* = .95 | *Time-points:*  *F(5,* 1967) = 1.31, *p* = .26 |
| T1 vs T3 | 0.04 | .14 | *t(*1967) = 0.26, *p* = .80 |  |
| T1 vs T4 | 0.03 | .17 | *t(*1967) = 0.16, *p* = .87 |  |
| T1 vs T5 | -0.06 | .19 | *t(*1967) = -0.33, *p* = .74 |  |
| T1 vs T6 | -0.42 | .21 | *t(*1967) = -1.95, *p* = .05 |  |
| **35-44 years old** |  |  |  |  |
| T1 vs T2 | 0.07 | .14 | *t(*1520) = 0.47, *p* = .65 | *Time-points:*  *F(5,* 1520) = 2.08, *p* = .07 |
| T1 vs T3 | 0.31 | .16 | *t(*1520) = 1.96, *p* = .05 |  |
| T1 vs T4 | 0.49 | .18 | *t(*1520) = 2.70, *p* = .007 |  |
| T1 vs T5 | 0.48 | .21 | *t(*1520) = 2.30, *p* = .02 |  |
| T1 vs T6 | 0.60 | .23 | *t(*1520) = 2.60, *p* = .009 |  |

|  | **Standardised beta** | **Std Error** | **T-test** | **Anova test for main effects** |
| --- | --- | --- | --- | --- |
| **45-54 years old** |  |  |  |  |
| T1 vs T2 | 0.04 | .16 | *t(*1159) = 0.28, *p* = .78 | *Time-points:*  *F(5,* 1159) = 1.90, *p* = .09 |
| T1 vs T3 | 0.31 | .19 | *t(*1159) = 1.63, *p* = .10 |  |
| T1 vs T4 | 0.10 | .22 | *t(*1159) = 0.05, *p* = .96 |  |
| T1 vs T5 | 0.38 | .24 | *t(*1159) = 1.60, *p* = .11 |  |
| T1 vs T6 | 0.58 | .25 | *t(*1159) = 2.27, *p* = .02 |  |
| **55-64 years old** |  |  |  |  |
| T1 vs T2 | 0.06 | .16 | *t(*1159) = 0.38, *p* = .71 | *Time-points:*  *F(5,* 1144) = 0.96, *p* = .44 |
| T1 vs T3 | -0.10 | .17 | *t(*1159) = -0.57, *p* = .57 |  |
| T1 vs T4 | -0.30 | .20 | *t(*1159) = -1.50, *p* = .13 |  |
| T1 vs T5 | -0.28 | .21 | *t(*1159) = -1.34, *p* = .18 |  |
| T1 vs T6 | -0.35 | .23 | *t(*1159) = -1.55, *p* = .12 |  |

|  | **Standardised beta** | **Std Error** | **T-test** | **Anova test for main effects** |
| --- | --- | --- | --- | --- |
| **65-74 years old** | | | | |
| T1 vs T2 | 0.27 | .23 | *t(*522) = 1.15, *p* = .25 | *Time-points:*  *F(5,* 522) = 1.39, *p* = .22 |
| T1 vs T3 | 0.49 | .26 | *t(*522) = 1.90, *p* = .06 |  |
| T1 vs T4 | 0.54 | .28 | *t(*522) = 1.90, *p* = .06 |  |
| T1 vs T5 | 0.74 | .30 | *t(*522) = 2.50, *p* = .01 |  |
| T1 vs T6 | 0.68 | .33 | *t(*522) = 2.08, *p* = .04 |  |
| **75-90 years old** |  |  |  |  |
| T1 vs T2 | 0.42 | .51 | *t(*131) = 0.83, *p* = .41 | *Time-points:*  *F(5,* 131) = 1.65, *p* = .15 |
| T1 vs T3 | 1.44 | .57 | *t(*131) = 2.53, *p* = .01 |  |
| T1 vs T4 | 0.40 | .63 | *t(*131) = 0.63, *p* = .53 |  |
| T1 vs T5 | 0.86 | .69 | *t(*131) = 1.25, *p* = .22 |  |
| T1 vs T6 | 1.38 | .77 | *t(*131) = 1.79, *p* = .08 |  |

| *Table S5. Positive link between adherence and wellbeing within demographic risk groups at T1. All models include adherence, age, gender, education, household status, works/study status and stringency of local measures as covariates. In addition, participants’ country of residence is entered as a random effect.* | | | |
| --- | --- | --- | --- |
|  | **Standardised beta** | **Std Error** | **T-test** |
| **Age: 16-24 year-olds** | | | |
| Low vs Medium adherence | 0.42 | .27 | *t(*1416) = 1.59, *p* = .11 |
| Low vs High adherence | 0.67 | .32 | *t(*1416) = 2.12, *p* = .03 |
| **Gender: Women** | | | |
| Low vs Medium adherence | 0.43 | .15 | *t(*4229) = 2.80, *p* = .005 |
| Low vs High adherence | 0.29 | .18 | *t(*4229) = 1.68, *p* = .09 |
| **Educational Attainment: None, primary and secondary** | | | |
| Low vs Medium adherence | 0.23 | .32 | *t(*1025) = 0.72, *p* = .47 |
| Low vs High adherence | 0.88 | .37 | *t(*1025) = 2.38, *p* = .02 |
| **Household status: Living alone** | | | |
| Low vs Medium adherence | 0.92 | .33 | *t(*731) = 2.77, *p* = .006 |
| Low vs High adherence | 0.77 | .39 | *t(*731) = 1.97, *p* = .05 |
| **Work/Study status: Inactive** | | | |
| Low vs Medium adherence | 0.14 | .32 | *t(*1060) = 0.45, *p* = .66 |
| Low vs High adherence | 0.90 | .37 | *t(*1060) = 2.50, *p* = .01 |

| *Table S6. Model predicting adherence (continuous values) from wellbeing (median split, categorical) within each time-point; combined with the results reported in the main text, these insignificant results indicate that adherence predicted wellbeing and not vice versa.* | | | |
| --- | --- | --- | --- |
|  | **Standardised beta** | **Std Error** | **T-test** |
| Wellbeing at T1 | 0.04 | .03 | *t(*6514) = 1.48, *p* = .14 |
| Wellbeing at T2 | 0.04 | .04 | *t(*1994) = 0.82, *p* = .41 |
| Wellbeing at T3 | 0.002 | .05 | *t(*1722) = 0.04, *p* = .97 |
| Wellbeing at T4 | -0.08 | .05 | *t(*1414) = -1.38, *p* = .17 |
| Wellbeing at T5 | 0.03 | .06 | *t(*1168) = 0.53, *p* = .59 |
| Wellbeing at T6 | -0.06 | .06 | *t(*1090) = -0.97, *p* = .33 |

# Replication of all analyses using continuous variables

| *Table S7. Demographic risk factors of wellbeing at T1 using continuous variables (Hypothesis 1)* | | | |
| --- | --- | --- | --- |
|  | **Standardised beta** | **Std Error** | **T-test** |
| Intercept | 17.96 | .35 | *t(*6518) = 57.37, *p* < .0001 |
| Age | 0.08 | .004 | *t(*6518) = 19.74, *p* < .0001 |
| Gender: men vs non-binary | -1.21 | .53 | *t(*6518) = -2.30, *p* = .02 |
| Gender: men vs not disclosed | -1.13 | .56 | *t(*6518) = -2.04, *p =* .04 |
| Gender: men vs woman | -0.65 | .11 | *t(*6518) = -5.98, *p* < .0001 |
| Household: solo vs cohabiting | 0.66 | .15 | *t(*6518) = 4.31, *p* < .0001 |
| Education | 0.25 | .08 | *t(*6518) = 3.44, *p =* .0004 |
| Work/study status: active vs inactive | -0.82 | .14 | *t(*6518) = -5.89, *p* < .0001 |
| Stringency of measures | -0.008 | .13 | *t(*6518) = -0.06, *p =* .95 |

| *Table S8. Demographic risk factors of wellbeing across time-points using continuous variables (Hypothesis 1)* | | | |
| --- | --- | --- | --- |
|  | **Standardised beta** | **Std Error** | **T-test** |
| Intercept | 17.96 | .35 | *t(*7730) = 57.37, *p* < .0001 |
| Age | 0.08 | .004 | *t(*6574) = 18.94, *p* < .0001 |
| Gender: non-binary | -1.23 | .58 | *t(*6574) = -2.12, *p* = .03 |
| Gender: not disclosed | -0.98 | .61 | *t(*6574) = -1.61, *p =* .11 |
| Gender: woman | -0.67 | .12 | *t(*6574) = -5.57, *p* < .0001 |
| Household: cohabiting | 0.60 | .15 | *t(*7730) = 3.98, *p* = .0001 |
| Education | 0.25 | .08 | *t(*6574) = 3.09, *p =* .002 |
| Work/study status: inactive | -0.75 | .14 | *t(*7730) = -5.42, *p* < .0001 |
| Stringency | -0.09 | .06 | *t(*7730) = -1.59, *p =* .11 |
| Time-points | 0.11 | .11 | *t(*7730) = 1.05, *p* = .29 |
| Time x Age | -0.002 | .001 | *t(*7730) = -1.77, *p* = .08 |
| Time x Gender: non-binary | -0.03 | .19 | *t(*7730) = -0.18, *p* = .86 |
| Time x Gender: not disclosed | -0.22 | .20 | *t(*7730) = -1.14, *p* = .25 |
| Time x Gender: woman | 0.05 | .04 | *t(*7730) = 1.19, *p* = .23 |
| Time x Household: cohabiting | -0.05 | .04 | *t(*7730) = -1.12, *p* = .26 |
| Time x Education | -0.0007 | .02 | *t(*7730) = -0.03, *p* = .98 |
| Time x Work/study status: inactive | 0.03 | .04 | *t(*7730) = 0.77, *p* = .44 |

| *Table S9. Disease vulnerability and wellbeing at T1 using continuous variables (Hypothesis 2)* | | | |
| --- | --- | --- | --- |
|  | **Standardised beta** | **Std Error** | **T-test** |
| Intercept | 21.06 | .35 | *t(*6516) = 60.76, *p* < .0001 |
| Self-vulnerability | -0.17 | .05 | *t(*6516) = -3.16, *p* = .002 |
| Others’ vulnerability | -0.15 | .05 | *t(*6516) = -2.80, *p* = .005 |
| Age | 1.24 | .06 | *t(*6516) = 19.97, *p* < .0001 |
| Gender: non-binary | -1.09 | .53 | *t(*6516) = -2.08, *p* = .04 |
| Gender: not disclosed | -1.05 | .56 | *t(*6516) = -1.89, *p =* .06 |
| Gender: woman | -0.59 | .11 | *t(*6516) = -5.43, *p* < .0001 |
| Household: cohabiting | 0.62 | .15 | *t(*6516) = 4.03, *p* = .0001 |
| Education | 0.26 | .07 | *t(*6516) = 3.47, *p =* .0005 |
| Work/study status: inactive | -0.82 | .14 | *t(*6516) = -5.89, *p* < .0001 |
| Stringency | -0.006 | .13 | *t(*6516) = -0.05, *p =* .96 |

| *Table S10. Disease vulnerability and wellbeing across time-points using continuous variables (Hypothesis 2)* | | | |
| --- | --- | --- | --- |
|  | **Standardised beta** | **Std Error** | **T-test** |
| Intercept | 20.99 | .34 | *t(*7733) = 62.05, *p* < .0001 |
| Self-vulnerability | -0.05 | .05 | *t(*7733) = -1.06, *p* = .29 |
| Others’ vulnerability | -0.07 | .05 | *t(*7733) = -1.39, *p* = .16 |
| Age | 1.20 | .06 | *t(*6574) = 20.92, *p* < .0001 |
| Gender: non-binary | -1.22 | .50 | *t(*6574) = -2.45, *p* = .01 |
| Gender: not disclosed | -1.26 | .54 | *t(*6574) = -2.33, *p =* .02 |
| Gender: woman | -0.56 | .10 | *t(*6574) = -5.35, *p* < .0001 |
| Household: cohabiting | 0.46 | .12 | *t(*7733) = 3.94, *p* = .0001 |
| Education | 0.25 | .07 | *t(*6574) = 3.51, *p =* .0005 |
| Work/study status: inactive | -0.68 | .11 | *t(*7733) = -6.38, *p* < .0001 |
| Stringency | -0.08 | .06 | *t(*7733) = -1.53, *p* = .13 |
| Time-points | 0.02 | .04 | *t(*7733) = 0.44, *p* = .66 |
| Time x Self-vulnerability | -0.03 | .02 | *t(*7733) = -2..07, *p* = .04 |
| Time x Others’ vulnerability | -0.002 | .02 | *t(*7733) = -0.15, *p* = .88 |

| *Table S11. Adherence to rules and wellbeing at T1 using continuous variables (Hypothesis 3)* | | | |
| --- | --- | --- | --- |
|  | **Standardised beta** | **Std Error** | **T-test** |
| Intercept | 21.07 | .35 | *t(*6517) = 60.60, *p* < .0001 |
| Adherence | 0.13 | .05 | *t(*6517) = 2.54, *p* = .01 |
| Age | 1.19 | .06 | *t(*6517) = 19.71, *p* < .0001 |
| Gender: non-binary | -1.21 | .53 | *t(*6517) = -2.08, *p* = .04 |
| Gender: not disclosed | -1.17 | .56 | *t(*6517) = -1.89, *p =* .06 |
| Gender: woman | -0.66 | .11 | *t(*6517) = -5.43, *p* < .0001 |
| Household: cohabiting | .064 | .15 | *t(*6517) = 4.03, *p* = .0001 |
| Education | 0.25 | .07 | *t(*6517) = 3.47, *p =* .0005 |
| Work/study status: inactive | -0.81 | .14 | *t(*6517) = -5.89, *p* < .0001 |
| Stringency | -0.01 | .13 | *t(*6517) = -0.05, *p =* .96 |

| *Table S12. Adherence to rules and wellbeing across time-points using continuous variables (Hypothesis 3)* | | | |
| --- | --- | --- | --- |
|  | **Standardised beta** | **Std Error** | **T-test** |
| Intercept | 21.22 | .33 | *t(*7735) = 63.99, *p* < .0001 |
| Adherence | 0.14 | .05 | *t(*7735) = 2.96, *p* = .003 |
| Age | 1.17 | .06 | *t(*6574) = 20.70, *p* < .0001 |
| Gender: non-binary | -1.29 | .50 | *t(*6574) = -2.58, *p* = .009 |
| Gender: not disclosed | -1.33 | .54 | *t(*6574) = -2.47, *p =* .01 |
| Gender: woman | -0.60 | .10 | *t(*6574) = -5.72, *p* < .0001 |
| Household: cohabiting | 0.48 | .12 | *t(*6574) = 4.12, *p* < .0001 |
| Education | 0.25 | .07 | *t(*6574) = 3.47, *p =* .0005 |
| Work/study status: inactive | -0.68 | .11 | *t(*6574) = -6.37, *p* < .0001 |
| Stringency | -0.09 | .06 | *t(*6517) = -1.58, *p =* .11 |
| Time | 0.02 | .04 | *t(*7735) = 0.49, *p* = .63 |
| Time x Adherence | -0.05 | .02 | *t(*7735) = -3.22, *p* = .001 |

# Aggregate mood variable

The aggregate mood variable comprised of the average of four single-item questions, which the participants evaluated on the same 5-point Likert scale as the WEMWBS, where 1= None of the time, 2= Rarely, 3= Some of the time, 4= Often, 5= All of the time. The items were: Over the last week, I have been feeling: (i) depressed, (ii) anxious, (iii) angry, (iv) lonely.

## Descriptive statistics

| *Table S13. Descriptive statistics of the variables reported in the main text at T1* | | | |
| --- | --- | --- | --- |
| **Continuous variables** | | | |
|  | **Range** | **Mean (SD)** | **Median** |
| **Wellbeing** | 7 – 35 | 21.56 (4.22) | 21.54 |
| **Self-vulnerability** | 1 – 100 | 46.41 (21.31) | 50.00 |
| **Others’ vulnerability** | 1 – 100 | 61.61 (15.62) | 61.54 |
| **Adherence: continuous** | 1 – 100 | 63.51 (21.57) | 56.00 |
| **Close circle compliance** | 0 – 99 | 14.71 (16.17) | 9.00 |
| **Country compliance** | 0 – 99 | 26.74 (23.12) | 21.00 |
| **Age** | 16 – 90 | 36.57 (14.25) | 33.00 |
| **Stringency** | 28 – 100 | 80.67 (9.78) | 80.00 |
| **Categorical variables (N’s)** | | | |
| **Adherence: categorical** | low = 1410, medium = 3553, high = 1712 | | |
| **Age** | 16–24 years = 1505, 25–34 years = 2093, 35–44 years = 1310,  45–54 years = 812, 55–64 years = 633, 65–74 years = 264,  75–90 years = 58 | | |
| **Gender** | man = 2204, woman = 4356, non-binary = 59, not disclosed = 56 | | |
| **Household** | solo = 818, cohabiting = 5857 | | |
| **Education** | none = 13, primary = 18, secondary = 1071, undergrad = 3096, postgrad = 2477 | | |
| **Work/study status** | active = 5532, inactive = 1143 | | |

| *Table S14. Descriptive statistics of each mood item at T1 and their pairwise correlations*  ^***^ *p* < .0001 | | | | |
| --- | --- | --- | --- | --- |
|  | **Depressed** | **Anxious** | **Angry** | **Lonely** |
| **Depressed** | 1.00 | 0.66^***^ | 0.53^***^ | 0.58^***^ |
| **Anxious** |  | 1.00 | 0.51^***^ | 0.46^***^ |
| **Angry** |  |  | 1.00 | 0.41^***^ |
| **Lonely** |  |  |  | 1.00 |
| **Range** | 1 – 5 | 1 – 5 | 1 – 5 | 1 – 5 |
| **Mean (SD)** | 2.69 (1.11) | 3.02 (1.10) | 2.62 (1.12) | 2.61 (1.24) |

| *Table S15. Mean wellbeing scores in the current study and previous studies in the literature.*  ^†^*Where long form of WEMWBS was used, the scores are halved to find the corresponding short WEMWBS score* | | | | | |
| --- | --- | --- | --- | --- | --- |
|  | **Overall** | **Men** | **Women** | **Young adults**  **(25–34 years)** | **Elderly adults**  **(75+ years)** |
| **Current study** | 21.6 | 22.3 | 21.4 | 20.9 | 22.3 |
| 1. **Gray et al. 2020**^†^   **(n = 12,554)** | 22.7 | 22.9 | 22.1 | 10.7 | 24.9 |
| 1. **Smith et al. 2020** | 20.8 | 21.5 | 20.6 | 19.3 | 22.8 |
| **Pre-pandemic population norm studies** | | | |  |  |
| 1. **Taggart et al. 2016** | 23.6 | 23.7 | 23.5 | n/a | n/a |
| 1. **ONS, Survey for Wales 2018-2019 (n = 11,922)** | 25.7 | 25.8 | 25.5 | 25.1 | 26.4 |
| 1. N. S. Gray *et al.*, The influence of the COVID-19 pandemic on mental well-being and psychological distress: Impact upon a single country. *Front. Psychiatry*. **11** (2020), , doi:10.3389/fpsyt.2020.594115. 2. ﻿L. Smith et al., Psychiatry Res. 291 (2020), doi:10.1016/j.psychres.2020.113138. 3. ﻿F. Taggart, S. Stewart-Brown, J. Parkinson, “Warwick-Edinburgh Mental Wellbeing Scale (WEMWBS) User Guide - Version 2” (2016), , doi:10.1007/978-3-319-91280-6_301816. 4. ﻿ONS, “National survey for Wales 2018-2019: Technical report” (2019). | | | | | |

## Replication of all analyses using the aggregate mood variable

| *Table S16. Demographic risk factors of the aggregate mood variable at T1 (Hypothesis 1)* | | | |
| --- | --- | --- | --- |
|  | **Standardised beta** | **Std Error** | **T-test** |
| Intercept | 2.70 | .07 | *t(*6514) = 38.57, *p* < .0001 |
| Age: 25-34 | 0.14 | .03 | *t(*6514) = 4.52, *p* < .0001 |
| Age: 35-44 | 0.35 | .04 | *t(*6514) = 9.92, *p* < .0001 |
| Age: 45-54 | 0.52 | .04 | *t(*6514) = 12.79, *p* < .0001 |
| Age: 55-64 | 0.65 | .04 | *t(*6514) = 14.58, *p* < .0001 |
| Age: 65-90 | 0.86 | .06 | *t(*6514) = 13.33, *p* < .0001 |
| Gender: non-binary | -0.26 | .12 | *t(*6514) = -2.29, *p* = .02 |
| Gender: not disclosed | -0.03 | .12 | *t(*6514) = -0.24, *p =* .81 |
| Gender: woman | -0.14 | .02 | *t(*6514) = -5.88, *p* < .0001 |
| Household: cohabiting | 0.20 | .03 | *t(*6514) = 6.01, *p* < .0001 |
| Education | 0.08 | .02 | *t(*6514) = 4.79, *p =* .0004 |
| Work/study status: inactive | -0.17 | .03 | *t(*6514) = -5.45, *p* < .0001 |
| Stringency | -0.02 | .02 | *t(*6514) = -0.73, *p =* .47 |

| *Table S17. Demographic risk factors of the aggregate mood variable across time-points using the (Hypothesis 1)* | | | |
| --- | --- | --- | --- |
|  | **Standardised beta** | **Std Error** | **T-test** |
| Intercept | 2.69 | .07 | *t(*7728) = 38.97, *p* < .0001 |
| Age: 25-34 | 0.19 | .03 | *t(*6570) = 5.32, *p* < .0001 |
| Age: 35-44 | 0.38 | .04 | *t(*6570) = 9.79, *p* < .0001 |
| Age: 45-54 | 0.57 | .04 | *t(*6570) = 12.85, *p* < .0001 |
| Age: 55-64 | 0.69 | .05 | *t(*6570) = 14.36, *p* < .0001 |
| Age: 65-90 | 0.89 | .07 | *t(*6570) = 13.43, *p* < .0001 |
| Gender: non-binary | -0.28 | .13 | *t(*6570) = -2.13, *p* = .03 |
| Gender: not disclosed | -0.05 | .14 | *t(*6570) = -0.38, *p =* .71 |
| Gender: woman | -0.13 | .03 | *t(*6570) = -4.85, *p* < .0001 |
| Household: cohabiting | 0.15 | .03 | *t(*7728) = 4.33, *p* < .0001 |
| Education | 0.08 | .02 | *t(*6570) = 4.78, *p* < .0001 |
| Work/study status: inactive | -0.15 | .02 | *t(*6570) = -6.41, *p* < .0001 |
| Stringency | 0.01 | .01 | *t(*6570) = 0.46, *p =* .65 |
| Time-points | 0.08 | .01 | *t(*7728) = 5.10, *p* < .0001 |
| Time x Age: 25-34 | -0.04 | .01 | *t(*7728) = -4.09, *p* < .0001 |
| Time x Age: 35-44 | -0.03 | .01 | *t(*7728) = -2.94, *p* = .003 |
| Time x Age: 45-54 | -0.05 | .01 | *t(*7728) = -4.03, *p* < .0001 |
| Time x Age: 55-64 | -0.04 | .01 | *t(*7728) = -4.15, *p* < .0001 |
| Time x Age: 65-90 | -0.04 | .01 | *t(*7728) = -2.81, *p* = .005 |
| Time x Gender: non-binary | -0.02 | .02 | *t(*7728) = -0.38, *p* = .70 |
| Time x Gender: not disclosed | -0.01 | .04 | *t(*7728) = -0.34, *p* = .73 |
| Time x Gender: woman | -0.004 | .008 | *t(*7728) = -0.46, *p* = .64 |
| Time x Household: cohabiting | -0.01 | .009 | *t(*7728) = -1.27, *p* = .20 |

| *Table S18. Disease vulnerability and the aggregate mood variable at T1 (Hypothesis 2)* | | | |
| --- | --- | --- | --- |
|  | **Standardised beta** | **Std Error** | **T-test** |
| Intercept | 3.05 | .07 | *t(*6516) = 42.77, *p* < .0001 |
| Self-vulnerability | -0.05 | .01 | *t(*6516) = -4.37, *p* < .0001 |
| Others’ vulnerability | -0.06 | .01 | *t(*6516) = -5.07, *p* < .0001 |
| Age | 0.28 | .01 | *t(*6516) = 20.94, *p* < .0001 |
| Gender: non-binary | -0.21 | .12 | *t(*6516) = -1.81, *p* = .07 |
| Gender: not disclosed | 0.004 | .12 | *t(*6516) = 0.03, *p =* .98 |
| Gender: woman | -0.12 | .02 | *t(*6516) = -4.99, *p* < .0001 |
| Household: cohabiting | 0.19 | .03 | *t(*6516) = 5.67 *p* = .0001 |
| Education | 0.07 | .02 | *t(*6516) = 4.54, *p =* .0005 |
| Work/study status: inactive | -0.19 | .03 | *t(*6516) = -6.23, *p* < .0001 |
| Stringency | -0.02 | .02 | *t(*6516) = -0.80, *p =* .42 |

| *Table S19. Disease vulnerability and the aggregate mood variable across time-points (Hypothesis 2)* | | | |
| --- | --- | --- | --- |
|  | **Standardised beta** | **Std Error** | **T-test** |
| Intercept | 3.1 | .07 | *t(*7733) = 47.66, *p* < .0001 |
| Self-vulnerability | -0.03 | .01 | *t(*7733) = -2.82, *p* = .005 |
| Others’ vulnerability | -0.04 | .01 | *t(*7733) = -3.58, *p* = .0003 |
| Age | 0.27 | .01 | *t(*6574) = 21.59, *p* < .0001 |
| Gender: non-binary | -0.26 | .11 | *t(*6574) = -2.40, *p* = .02 |
| Gender: not disclosed | -0.05 | .12 | *t(*6574) = -0.40, *p =* .68 |
| Gender: woman | -0.12 | .02 | *t(*6574) = -5.16, *p* < .0001 |
| Household: cohabiting | 0.11 | .03 | *t(*7733) = 4.31 *p* < .0001 |
| Education | 0.06 | .02 | *t(*6574) = 4.20, *p* < .0001 |
| Work/study status: inactive | -0.16 | .02 | *t(*7733) = -6.97, *p* < .0001 |
| Stringency | 0.005 | .01 | *t(*7733) = 0.43, *p =* .67 |
| Time-points | 0.03 | .008 | *t(*7733) = 3.44, *p* = .0006 |
| Time x Self-vulnerability | -0.006 | .003 | *t(*7733) = -1.77, *p* = .08 |
| Time x Others’ vulnerability | 0.001 | .003 | *t(*7733) = 0.40 *p* = .69 |

| *Table S20. Adherence to rules and the aggregate mood variable at T1 (Hypothesis 3)* | | | |
| --- | --- | --- | --- |
|  | **Standardised beta** | **Std Error** | **T-test** |
| Intercept | 3.04 | .07 | *t(*6517) = 42.49, *p* < .0001 |
| Adherence | -0.01 | .01 | *t(*6517) = -0.84, *p* = .40 |
| Age | 0.27 | .01 | *t(*6517) = 20.39, *p* < .0001 |
| Gender: non-binary | -0.25 | .11 | *t(*6517) = -2.15, *p* = .03 |
| Gender: not disclosed | -0.02 | .12 | *t(*6517) = -0.19, *p =* .85 |
| Gender: woman | -0.14 | .02 | *t(*6517) = -5.80, *p* < .0001 |
| Household: cohabiting | 0.21 | .03 | *t(*6517) = 6.19, *p* < .0001 |
| Education | 0.07 | .02 | *t(*6517) = 4.49, *p* < .0001 |
| Work/study status: inactive | -0.19 | .03 | *t(*6517) = -6.17, *p* < .0001 |
| Stringency | -0.02 | .02 | *t(*6517) = -0.78, *p =* .44 |

| *Table S21. Adherence to rules and the aggregate mood variable across time-points (Hypothesis 3)* | | | |
| --- | --- | --- | --- |
|  | **Standardised beta** | **Std Error** | **T-test** |
| Intercept | 3.10 | .07 | *t(*7735) = 47.36, *p* < .0001 |
| Adherence | 0.001 | .01 | *t(*7735) = 0.13, *p* = .90 |
| Age | 0.26 | .01 | *t(*6574) = 20.88, *p* < .0001 |
| Gender: non-binary | -0.29 | .11 | *t(*6574) = -2.64, *p* = .008 |
| Gender: not disclosed | -0.07 | .12 | *t(*6574) = -0.59, *p =* .56 |
| Gender: woman | -0.13 | .02 | *t(*6574) = -5.75, *p* < .0001 |
| Household: cohabiting | 0.12 | .03 | *t(*6574) = 4.79, *p* < .0001 |
| Education | 0.07 | .02 | *t(*6574) = 4.26, *p* < .0001 |
| Work/study status: inactive | -0.16 | .02 | *t(*6574) = -6.93, *p* < .0001 |
| Stringency | -0.005 | .01 | *t(*6517) = 0.46, *p =* .64 |
| Time | 0.03 | .008 | *t(*7735) = 3.63, *p* = .0003 |
| Time x Adherence | -0.008 | .003 | *t(*7735) = -2.54, *p* = .01 |
